# Trapping DNA Replication Origins from the Human Genome

**DOI:** 10.3390/genes4020198

**Published:** 2013-04-17

**Authors:** Toshihiko Eki, Yasufumi Murakami, Fumio Hanaoka

**Affiliations:** 1 Molecular Genetics Laboratory, Division of Bioscience and Biotechnology, Department of Environmental and Life Sciences, Toyohashi University of Technology, 1-1 Hibarigaoka, Tempaku-cho, Toyohashi, Aichi 441-8580, Japan; 2 Cellular Physiology Laboratory, RIKEN, 2-1 Hirosawa, Wako, Saitama 351-0198, Japan; 3 Department of Biological Science and Technology, Faculty of Industrial Science and Technology, Tokyo University of Science, 2641 Yamazaki, Noda, Chiba 278-8510, Japan; E-Mail: yasufumi@rs.noda.tus.ac.jp; 4 Faculty of Science, Gakushuin University, 1-5-1 Mejiro, Toshima-ku, Tokyo 171-8588, Japan; E-Mail: fumio.hanaoka@gakushuin.ac.jp

**Keywords:** DNA replication, replication origins, sequence analysis, human genome, competitive PCR

## Abstract

Synthesis of chromosomal DNA is initiated from multiple origins of replication in higher eukaryotes; however, little is known about these origins’ structures. We isolated the origin-derived nascent DNAs from a human repair-deficient cell line by blocking the replication forks near the origins using two different origin-trapping methods (*i.e.*, UV- or chemical crosslinker-treatment and cell synchronization in early S phase using DNA replication inhibitors). Single-stranded DNAs (of 0.5–3 kb) that accumulated after such treatments were labeled with bromodeoxyuridine (BrdU). BrdU-labeled DNA was immunopurified after fractionation by alkaline sucrose density gradient centrifugation and cloned by complementary-strand synthesis and PCR amplification. Competitive PCR revealed an increased abundance of DNA derived from known replication origins (*c-myc* and *lamin* B2 genes) in the nascent DNA fractions from the UV-treated or crosslinked cells. Nucleotide sequences of 85 and 208 kb were obtained from the two libraries (I and II) prepared from the UV-treated log-phase cells and early S phase arrested cells, respectively. The libraries differed from each other in their G+C composition and replication-related motif contents, suggesting that differences existed between the origin fragments isolated by the two different origin-trapping methods. The replication activities for seven out of 12 putative origin loci from the early-S phase cells were shown by competitive PCR. We mapped 117 (library I) and 172 (library II) putative origin loci to the human genome; approximately 60% and 50% of these loci were assigned to the G-band and intragenic regions, respectively. Analyses of the flanking sequences of the mapped loci suggested that the putative origin loci tended to associate with genes (including conserved sites) and DNase I hypersensitive sites; however, poor correlations were found between such loci and the CpG islands, transcription start sites, and K27-acetylated histone H3 peaks.

## 1. Introduction

DNA replication is a fundamental process for maintaining and transmitting genetic information to proliferating cells. In eukaryotes, genomic DNA replication starts bi-directionally from sites called “replication origins”. Because the initiation step of DNA replication is crucial for regulation of cell proliferation, the structures of the origins and the proteins involved in their functions have been extensively studied. In *Saccharomyces cerevisiae*, where DNA replication has been studied in detail, the nucleotide sequence motifs that are conserved in the replication origins and are called autonomously replicating sequences (ARSs) have been elucidated. In this species, the origin recognition complex (ORC) and the proteins associated with it have been shown to play essential roles in origin replication initiation. The ORC specifically recognizes a 17-bp AT-rich consensus sequence (ARS consensus sequence, ACS), where the initiation proteins assemble in a stepwise manner to initiate DNA replication [1]. In fission yeast, the distinct core sequences of the origins have not been identified although it is known that the origins in *S. pombe* contain AT-rich regions [2,3,4,5]. In metazoans, the individual origins of replication within, for example, the Chinese hamster *DHFR* locus [6,7] human *c-myc* [8,9], and *lamin* B2 genes [10] have been extensively studied. Although some of these studies [8,9] suggested that DNA replication initiates from a broad region (*i.e.*, an initiation zone) in the genome, no particular sequences were identified. Microarray- and high throughput sequencing-mediated methods have been recently applied to map the replication origins in the sequenced mammalian genomes (including the human genome) [11,12,13,14,15,16,17,18,19] (also reviewed in [20,21]). These genome-wide mapping studies on replication initiation sites have identified a number of replication origins in mammals. Some of these studies have suggested that the human origins preferentially associate with relatively G+C-rich regions [11,19] (unlike that observed with yeast origins), and CpG islands and transcription levels near the origins can influence the replication initiation events [12,15,16]; this suggests potential regulation of replication initiation coupled with transcriptional regulation during development and differentiation [13,18,22,23,24]. Although one study has claimed that a 36-bp human consensus sequence supports autonomous DNA replication [25], we still do not know if any conserved sequences or motifs encode replication origins. In addition, studies conducted some time ago have suggested that replication initiation sites are closely associated with the nuclear structure [26,27,28,29] (also reviewed in [30]) of the cell; however, little is known about the distribution of nuclear attachment sequences around the origins, such as the nuclear scaffold associated region (SAR) and the nuclear matrix associated region (MAR).

Over the past decades, several methods for origin mapping have been developed for identifying replication origins in eukaryotic chromosomes [20,31]. These methods fall into three categories: (1) analysis of nascent DNA (e.g., isolation of newly replicated DNA); (2) analysis of DNA structure (e.g., 2D-gel electrophoresis analysis or bubble trap [32]); and (3) ARS assays on cellular DNA. With one exception [25], the difficulty involved in conducting ARS assays in mammalian cells has resulted in previous studies on origins being performed mainly by nascent DNA analyses or structural analysis of DNA. Since the former methods are suitable for detecting unidentified origins, unlike a gel electrophoresis-based analysis, a nascent DNA-based approach has been used for recent comprehensive studies on replication origins [11,12,13,14,15,16,18,19]. In analyses of nascent DNA molecules, the bromodeoxyuridine (BrdU)-labeled DNAs and the λ exonuclease-resistant short DNAs (*i.e.*, nascent DNA with a 5' RNA primer) from the replication origins have frequently been used to assay for origin activities and/or clone such DNAs from the origins. Both methods have their drawbacks, which include contamination of nicked BrdU-labeled DNA in the former method, and variable efficiency of λ exonuclease activity and RNase contamination in the latter. Nevertheless, Karnani *et al.* compared both methods and showed that their usefulness for mapping replication origins in human cells was comparable [14]. In the former method, when exponentially proliferating (log-phase) cells were labeled with BrdU, vast amounts of BrdU-labeled DNAs were generated; these were distinct from the origins themselves, and were likely to be broken during purification leading to contamination of the BrdU-labeled short DNAs produced from the origins. Therefore, when analyzing nascent DNA for mapping and cloning replication origins using the BrdU-labeling method, it is very important to effectively arrest the replication fork movements near the origins upon labeling with BrdU. In other words, the ability to efficiently arrest replication forks near their origins is crucial for origin mapping using BrdU-labeled nascent DNAs.

In this study, our intention was to systematically clone the BrdU-labeled nascent DNAs from arrested replication forks that had arrested near the origins and map the replication origins in the human genome at the nucleotide sequence level in order to elucidate the features of the origin sequences. To achieve this, first, we developed a novel origin-trapping method by forming UV-induced DNA lesions in a nucleotide excision repair-deficient human (GM8207) cell line. Replication fork progression was effectively blocked in the exponentially proliferating GM8207 cells by UV irradiation and Trioxsalen-mediated DNA crosslinking. We performed competitive PCR assays using the nascent DNAs from the treated cells and showed that DNAs from well-characterized *c-myc* and *lamin* B2 origins were enriched by the UV- and crosslink-mediated origin-trapping methods. Second, for isolating the early-firing origins from highly synchronized cells, we labeled the nascent DNAs with BrdU in early S phase in the presence of replication inhibitors.

BrdU-labeled DNAs prepared by the two different methods were cloned for construction of genomic DNA libraries (*i.e.*, library I from the UV-irradiated log-phase cells and library II from the cells synchronized in early S phase) and for their sequence analyses. In particular, the nascent DNA abundance of 12 potential origin loci identified from the synchronized cells was examined by competitive PCR to access the origin activities of these loci from library II.

The nucleotide sequences from libraries I (85 kb) and II (208 kb) were compared with each other or with control sequences from the human genome to elucidate the features of the isolated putative origins. The results of this analysis indicated that the two libraries differed in their G+C composition and their replication-related motif contents. One hundred and seventeen and 172 putative origin loci from libraries I and II, respectively, were mapped to the human genome. In addition, the flanking sequences of the mapped origin loci were analyzed by comparison to 236 genomic control loci to examine the association of the origin loci with genes, DNase I hypersensitive sites, transcription start sites, CpG islands and an epigenetic marker of K27-acetylated histone H3 peaks.

Although our comprehensive study has been conducted on a relatively small scale, the origin-trapping methods reported here could be useful for studying DNA replication origins in a number of eukaryotes whose genomes have not been sequenced. That the methods require little or no specialist genome analysis tools (such as microarrays or next generation sequencers) should be a distinct advantage for many researchers.

## 2. Results and Discussion

### 2.1. Scheme for Isolation and Cloning of Nascent DNAs from Origins of Replication

**Figure 1:**
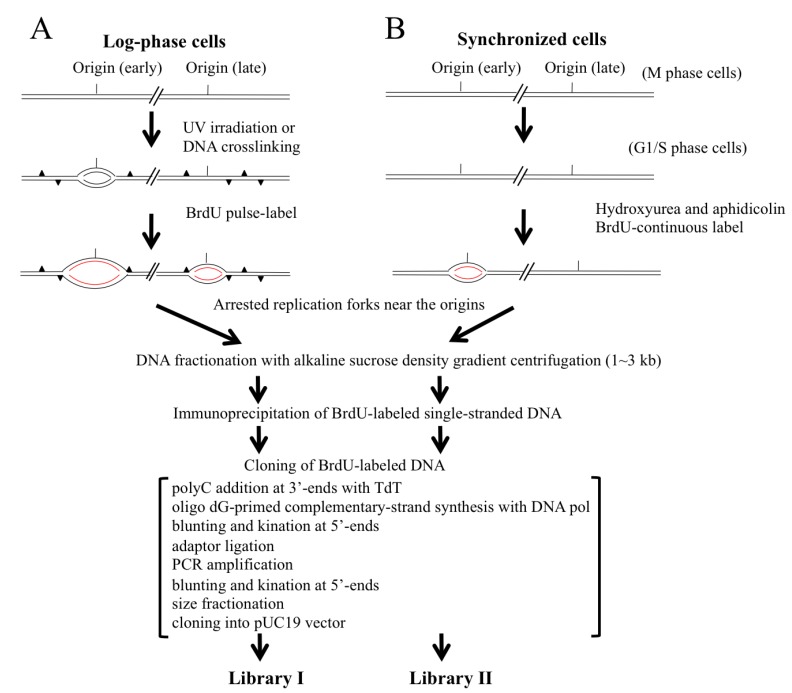
Scheme for trapping and isolating nascent DNAs from origins of replication in human cells. (**A**) The origin-trapping method where UV- and chemical crosslinking-mediates replication fork arrest in exponentially proliferating cells; (**B**) The method for trapping the early-initiating origins is conducted by arresting the cell populations in early S phase using replication inhibitors. BrdU-labeled nascent DNAs from the origins are shown in red.

In this study, we designed and used two different methods for isolating the nascent DNA molecules arising from DNA replication origins (Figure 1). The approach shown in Figure 1A depends upon arresting the replication fork movements near the origins by forming DNA adducts with UV irradiation in DNA synthesizing cells from a proliferating cell population. To evaluate this approach, we also employed the origin-trapping method by using a chemical crosslinker reported in the literature previously [33,34]. In parallel experiments, we aimed to facilitate accumulation of the nascent DNAs around the origins firing in early S phase by arresting the replication forks with replication inhibitors (Figure 1B). Synchronized M phase cells were allowed to progress to early S phase and were arrested using DNA synthesis inhibitors (*i.e.*, aphidicolin and hydroxyurea) and then labeled with BrdU to allow the nascent DNAs to be BrdU-labeled in the replication forks that were arrested near the early-firing origins. Nascent DNAs from origins initiating in early and late S phase could be trapped by the former method, whereas DNAs from the early-replicating origins were mainly isolated by the latter method. Trapped BrdU-incorporated nascent DNAs near the origins can be completely purified by alkaline sucrose density gradient centrifugation followed by immunoprecipitation with an anti-BrdU antibody and protein A/G-coated latex beads. To clone a very small amount of BrdU-labeled single-stranded DNA, the purified single-stranded DNA was converted into double-stranded DNA via tailing with poly dC at the 3'-ends by terminal deoxynucleotidyl transferase (TdT) and oligo dG-primed DNA synthesis of the complementary strands by DNA polymerase. After adaptor ligation, the DNA was PCR amplified and size fractionated by agarose gel electrophoresis for cloning into a plasmid. In this study, libraries I and II were constructed from the nascent DNAs isolated from UV-irradiated log-phase and synchronized cells, respectively (Figure 1).

### 2.2. Trapping Nascent DNAs in Human Cells by UV Irradiation and Chemical Crosslinking

**Figure 2:**
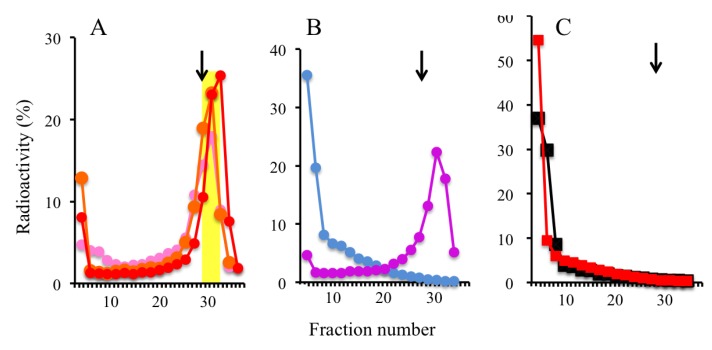
Inhibition of DNA synthesis in GM8207 cells after UV irradiation. (**A**) Distribution of pulse-labeled nascent DNAs in the UV (300 J/m^2^)-irradiated cells after a 0-min (red), 30-min (orange), or 90-min (pink) chase along the alkaline sucrose density gradient fractions; (**B**) Distribution of pulse-labeled nascent DNA in the non-irradiated cells after a 0-min (purple), or a 90-min (light blue) chase; (**C**) Distribution of pre-labeled parental DNA with [^14^C] TdR in the gradient fractions at 45 min after UV-irradiation (0: black; 300 J/m^2^: red). Arrows: 3 kb size marker.

We first applied the UV-mediated origin-trapping method to the human SV40-transformed fibroblast cell line GM8207, which is deficient in nucleotide excision repair [35]. In GM8207 cells after UV irradiation, recovery from the arrested replication fork movement by DNA repair mechanisms is poor unlike in other human cell lines such as HeLa cells (data not shown). In addition, GM8207 cells are suitable for isolation of nascent DNA molecules, because BrdU-incorporation into UV-damaged genomic DNAs by DNA repair mechanisms can be minimized. We examined the effects of UV irradiation during cellular DNA synthesis by pulse-chase experiments followed by analytical alkaline sucrose density gradient centrifugation. DNA (approximately 0.5–2 kb) was labeled with [^3^H]thymidine (TdR) during a 15-min pulse in GM8207 cells synchronized in S phase, after which the labeled DNA was allowed to maturate to bulk DNA during the 90-min chase in the non-irradiated cells (Figure 2B). However, DNA elongation was totally inhibited in the cells treated with 300 J/m^2^ UV prior to pulse-labeling, as was expected (Figure 2A). To examine if genomic DNAs are broken by UV irradiation, the cells whose parental DNA strands were uniformly labeled with [^14^C]TdR were synchronized in S phase and irradiated with or without 300 J/m^2^ UV. After 45 min in culture, labeled DNAs were observed in the bulk DNA fractions indicating that the DNA strand breakage after UV irradiation was negligible (Figure 2C). BrdU-labeled DNA of approximately 0.5–2 kb was pooled for further DNA purification (yellow zone in Figure 2A).

We also compared the previously reported DNA crosslink-mediated origin-trapping method that uses Trioxsalen [33,34] with our UV-mediated method. Inter-crosslinking of double-stranded DNA also effectively arrests the progression of replication forks as well as UV-induced DNA adducts on the template DNA for replication. Dimitrova *et al.* [34] isolated and cloned nascent DNAs from mouse cells by Trioxsalen-mediated crosslinking and showed that some of the isolated fragments were A+T-rich and were bound to protein factors. Although these researchers showed enrichment of the ARS sequences from yeast cells using this method, it is not known if the mouse origins were concentrated by the method, or if the isolated DNAs acted as origins *in vivo*.

**Figure 3:**
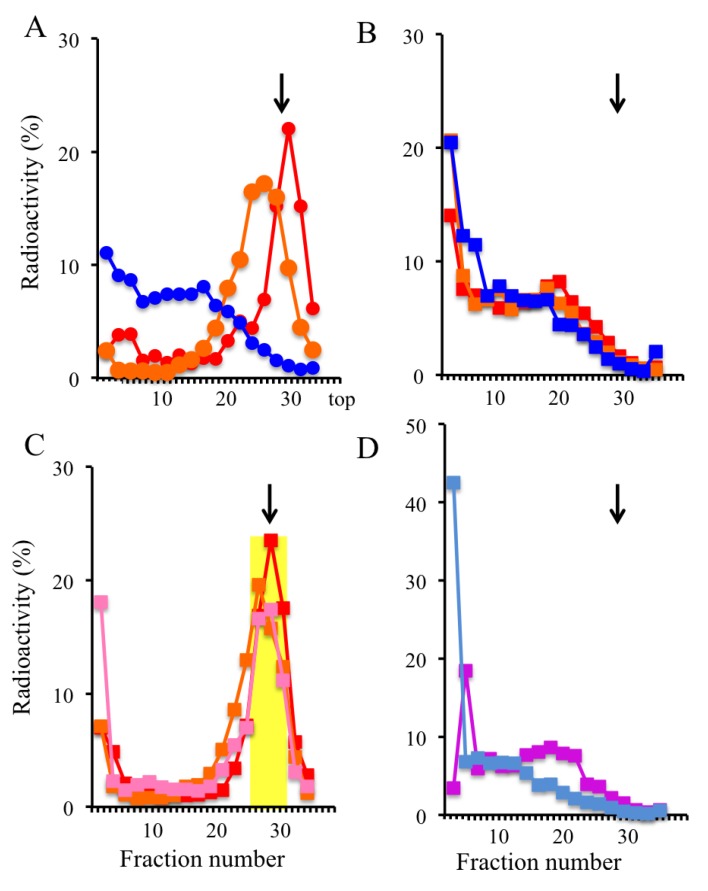
Inhibition of DNA synthesis in GM8207 cells by Trioxsalen treatment. (**A**) Distribution of pulse-labeled nascent DNA in the cells treated with Trioxsalen (photo-irradiation conditions: 3 min, twice in red; 2 min, once in orange; no irradiation in blue) in the alkaline sucrose density gradient fractions; (**B**) Distribution of pulse-labeled nascent DNA in the cells without Trioxsalen; (**C**) Distribution of pulse-labeled nascent DNA in the cells crosslinked with Trioxsalen (3 min, twice) after 0-min (red), 30-min (orange), and a 90-min (pink) chase; (**D**) Distribution of pulse-labeled nascent DNA in the non-crosslinked cells after 0-min (purple) and 90-min (light blue) chase. Arrows: 3 kb size marker.

We initially examined the inhibitory effects of Trioxsalen treatment (*i.e.*, variations in the times used for irradiation for photo-crosslinking) on DNA synthesis in GM8207 cells. The cells in mid-S phase were photo-irradiated using the conditions indicated in the Experimental Section, with or without Trioxsalen (1.25 μg/mL) (Figure 2). The sizes of the pulse-labeled DNAs were >10 kb in the irradiated and non-irradiated cells without Trioxsalen (Figure 3B), and in the non-irradiated cells with Trioxsalen (blue line in Figure 3A); however, a labeled nascent DNA peak of approximately 3 kb was observed from the cells irradiated with Trioxsalen (3 min, twice) (red line in Figure 3A). As shown in Figure 3C, nascent DNA formation was arrested at around 3 kb, even after a 90-min chase in the Trioxsalen-treated cells. In contrast, labeled DNA was found to maturate to bulk DNA in the non-treated cells after the chase (Figure 3D). The peak fractions of the BrdU-labeled DNAs (approximately 1–4 kb) were pooled for additional DNA purification and use in nascent DNA abundance assays (yellow zone in Figure 3C).

### 2.3. Trapping Newly Synthesized DNAs in Cells Arrested in Early S Phase with Aphidicolin and Hydroxyurea

**Figure 4:**
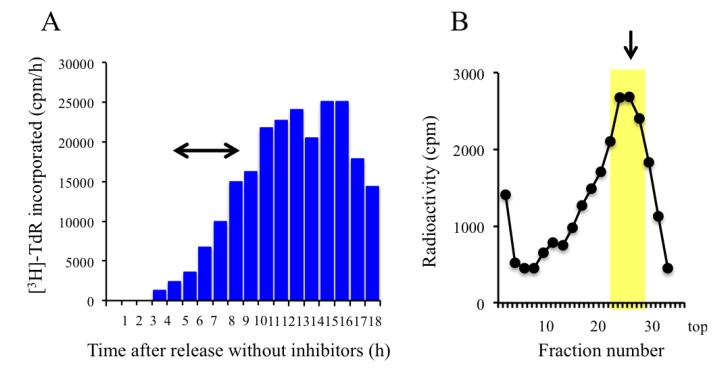
Analyses of DNA synthesis in GM8207 cells synchronized in early S phase. (**A**) DNA synthesis in the synchronized cells in the absence of aphidicolin and hydroxyurea after release from the mitotic block. [^3^H]TdR incorporated into the cells was measured at 1-h intervals after release. Arrowed bar: labeling period with BrdU. Labeling was carried out in the presence of aphidicolin and hydroxyurea; (**B**) Distribution of labeled DNA molecules in the fractions obtained from alkaline sucrose density gradient centrifugation. Arrow: 3 kb marker.

To identify the early-initiating origins in the genome, we isolated nascent DNAs from GM8207 cells arrested in early S phase after double cell synchronization (Figure 1B). After release from the aphidicolin block, cells that had been synchronized in M phase with colcemide were released and allowed to enter G1, after which they were continuously labeled with BrdU in the presence of aphidicolin and hydroxyurea during early-S phase. Under these conditions, the replication forks in most of the cells would be arrested near their replication origins. To monitor DNA synthesis in the cell population after release from the mitotic block, [^3^H]TdR incorporated into the cells and measured in the absence of the replication inhibitors at 1-h intervals after release (Figure 4A), showed that the synchronized population of cells started to replicate their DNA from about 5 h post-release. To isolate the nascent DNA, the synchronized cells were continuously labeled with BrdU in the presence of high concentrations of aphidicolin (5 μg/mL) and hydroxyurea (1.5 mM) from 5 h to 9 h after release, after which the labeled DNA was purified. The results of the analytical alkaline sucrose density gradient centrifugation showed that nascent DNA of approximately 3–5 kb had accumulated in the cells that were arrested in early S phase (Figure 4B). The labeled DNA peak in the gradient fractions was found at a position that was slightly larger than the 0.5–3 kb observed in the UV-treated or crosslinked cells. The size difference of the nascent DNA observed in the UV-radiated and synchronized cells may be related to the different modes of action of the treatments used for replication fork arrest because DNA crosslinking arrests replication forks more tightly than treatment with DNA synthesis inhibitors. Nascent DNA from the peak fractions was pooled for cloning (yellow zone in Figure 4B). The mean sizes of the DNA inserts in the libraries prepared from the UV-irradiated and synchronized cells differed from each other (Section 2.5); this probably reflects the different sizes of the pooled DNA obtained from the different treatments of the cells.

### 2.4. Isolation of Nascent DNAs and Evaluation of Enrichment of the Origin-Derived DNAs Using Competitive PCR

To isolate the BrdU-labeled DNA from the UV irradiated cells, we used alkaline sucrose density gradient centrifugation and immunoprecipitation with an anti-BrdU antibody, according to a previously reported procedure [34] with the following modification (*i.e.*, use of protein A/G-coated latex beads for minimizing non-specific binding of single-stranded DNA to the beads). We evaluated the purification coefficient of the nascent DNA from the GM8207 cells treated with Trioxsalen (3 min, one treatment). Parental strands from the bulk DNA were uniformly labeled with [^14^C]TdR, while the nascent DNAs were labeled with BrdU and [^3^H]cytidine. After fractionation by alkaline sucrose density gradient centrifugation, the nascent DNAs arrested at around 3 kb were pooled and purified by immunoprecipitation. The total radioactivity counts from the [^3^H] and [^14^C] recovered in each step are summarized in Table 1. The results indicate that the nascent DNA fragments were concentrated more than 3,000-fold in the immunopurified fraction.

**Table 1:** Purification of BrdU-labeled nascent DNAs.

Fraction	^3^H total count (cpm)	^14^C total count (cpm)	^3^H/^14^C ratio	^3^H-labeled DNA abundance (fold)
Total DNA	12,997	127,230	0.102	1
Pooled DNA	4,811	293	16.4	161
Immunopurified DNA	3,320	<10	>332	>3,250

**Figure 5:**
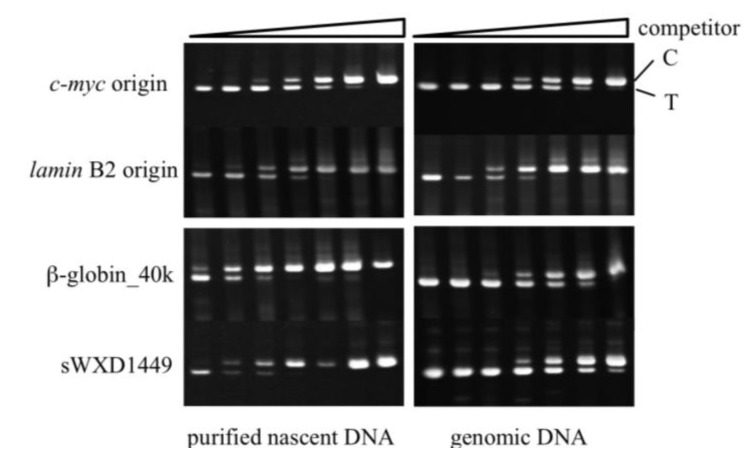
Competitive PCR assay for assessing the relative abundance of DNA from *c-myc*, *lamin* B2, β-globin_40k, and sWXD1449 loci. Competitive assays for the four loci (indicated on the left of the figure) were carried out using the purified BrdU-labeled DNA fractions from the crosslinked cells (left columns) and genomic DNA (right columns) as a template in the presence of the indicated number of the corresponding competitor DNA molecules (from left to right at the top of the lanes: 40, 130, 400, 1,200, 3,600, 10,800, and 32,400 molecules). PCR products derived from target DNA (T) and competitor DNA (C) are indicated. The product from the competitor DNA is 20 bp longer than the target DNA-derived product. The corresponding results from quantitation of the PCR products are shown in Figure 6C and supplementary Table S2.

**Figure 6:**
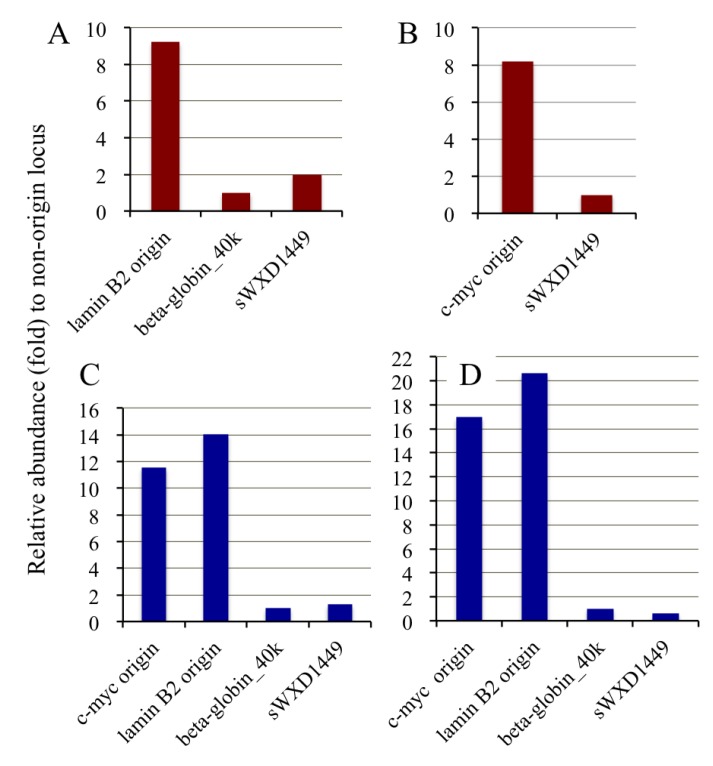
Abundance of the origin-derived DNAs in the DNA fractions purified from UV irradiated and crosslinked cells. The relative DNA abundances from the origin and non-origin loci compared with the β-globin_40k (**A**, **C**, **D**) or the sWXD1449 (**B**) locus in the purified BrdU-labeled DNA fractions from UV-irradiated (**A** and **B**) or crosslinked (**C** and **D**) cells are shown. Further details appear in supplementary Table S2.

We also examined enrichment of the nascent DNAs from the known origins of replication using competitive PCR methodology [36]. Replication origins in the human *c-myc* [8] and *lamin* B2 [10] loci have been extensively studied and can, therefore, be used as positive controls. Two non-origin loci were also selected for the assay. One locus (β-globin_40k) is a site in the human β-globin gene locus that is approximately 20 kilobases from the replication initiation zone [37,38]. The other is the sWXD1449 locus (accession no. L77324) that is located on human chromosome Xq27; this region has been shown to be replicated during the very late stages of S phase by Hansen *et al.* [39]. Primer sets for the four loci (*i.e.*, *c-myc*, *lamin* B2, β-globin_40k, and sWXD1449) were prepared for competitive PCR (supplementary Table S1) and the abundance of the DNAs from each locus was determined for both the nascent and genomic DNA fractions. For nascent DNA abundance assays, the adjacent DNA sequences to the origins were frequently used as negative controls. Several primer sets for the non-origin loci including the flanking regions of *c-myc* and *lamin* B2 origins have been tested, however, we could only successfully prepare two primer sets for two non-origin loci (β-globin_40k and sWXD1449) and competitors that had sufficient stability for use in the competitive PCR assay. In this assay, the relative abundance of DNA from an origin locus in the nascent DNA, relative to a genomic DNA locus, was higher than that for a non-origin locus. PCR amplicons from the competitive PCR assays were separated by gel electrophoresis (Experimental Section); the results of which are shown in Figure 5 and summarized in Figure 6 and supplementary Table S2. The relative abundances of the origin-derived DNAs in the purified nascent DNA fractions from the UV-irradiated (Figure 6A,B) and crosslinked (Figure 6C,D) cells were approximately 8–20-fold higher than those from the non-origin loci-derived DNAs; this indicated that the nascent DNAs from the origins were enriched in the DNA fractions purified from the UV- and crosslinker-treated cells. Thus, the origin-trapping methods by UV irradiation and chemical crosslinking are effective at isolating replication origins from human cells.

### 2.5. Cloning of BrdU-Labeled Nascent DNA and Sequence Analysis of the DNA Clones

Based on the procedures described above, we have independently isolated and cloned BrdU-labeled nascent DNAs from UV (300 J/m^2^)-irradiated proliferating GM8207 cells and cells synchronized in early S phase. Because the quantity of the labeled nascent DNA was very small, and BrdU-labeled DNA is both heat- and photo-labile (e.g., heat denaturation at 95 °C for 5 min completely disrupted the BrdU-labeled DNAs), we avoided exposing the DNA samples to high temperatures and light during the cloning process. Nascent DNAs were cloned into plasmids via addition of poly dC tails at their 3' ends, oligo dG-primed complimentary strand synthesis, adaptor ligation, and PCR amplification (as described in the Experimental Section). Complementary strand synthesis by DNA polymerase was totally dependent upon both the poly dC tailing reaction and the oligo dG primer (data not shown). For DNA cloning from the UV-irradiated samples, we used T7 DNA polymerase for complementary strand synthesis at 37 °C; however, the resultant clones frequently contained non-specifically primed DNA inserts that were presumably caused by the repetitive sequences in the DNA samples. Therefore, despite the need to avoid using a high temperature reaction (65 °C for 4 min), we used Taq DNA polymerase for DNA synthesis for cloning the DNA samples from the synchronized cells. In addition, we implemented a size fractionation process for the amplified DNAs to remove the contamination derived from clones with short inserts. In this study, the two genomic libraries (I and II) were prepared from the UV-irradiated proliferating cells and the cells arrested in early S phase, respectively.

By subsequent sequencing of the DNA inserts in both directions, a large number of nucleotide sequences were obtained from each of the libraries, library I (133 sequences) and II (221 sequences) (Table 2; supplementary Table S3). Clones from library II contained longer DNA inserts than those from library I (Table 2, 640 bp *versus* 941 bp), which was made from the UV-irradiated cells; this result is consistent with the sizes of the nascent DNAs (0.5–2 kb in Figure 2A *versus* 3–5 kb in Figure 4B) that were used for library preparation, although we cannot exclude the possibility that a size bias occurred during the cloning process. Library I contained more chimeric clones than library II (23 *versus* 7 out of the total number of clones analyzed in each library).

**Table 2:** Characteristics of the sequenced clones from libraries I and II.

	Library I	Library II
DNA source	UV-treated proliferating cells	Synchronized cells in early S phase
No. of determined sequences	133	221
No. of independently sequenced inserts	132	187
Total length of the sequences (bp)	85,168	207,895
Mean length of the sequences (bp)	640 ± 328	941 ± 279

Note: Number of independently sequenced inserts: this denotes where two independently sequenced DNA inserts in a chimeric clone were counted as two, and two sequences from a single DNA insert (e.g., two sequences reads in both direction for a long-insert DNA) were counted as one. The mean length for each of the library sequences is shown with the associated standard deviation.

### 2.6. Competitive PCR Assays for Library II Loci

**Figure 7:**
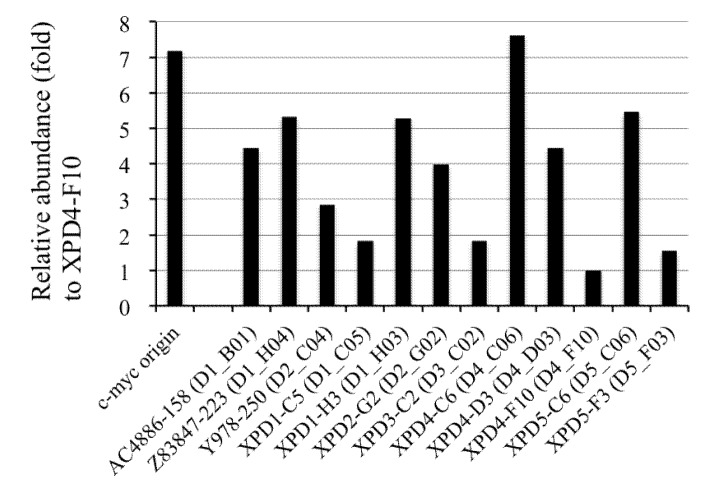
Measurement of the relative abundances of the nascent DNAs from 12 library II loci using competitive PCR assays. The DNA abundance for each locus relative to that of XPD4-F10 is indicated. The locus name and the derived sequence ID (in parentheses) are shown. Details of the results appear in supplementary Table S4.

We next examined the abundance of each locus identified in the nascent DNAs using competitive PCR. This enabled us to examine if individual loci in the cloned DNAs were derived from an origin region. For this purpose, 107 sets of PCR primers were designed based on unique sequences in library II. After screening to obtain unique signals in both the PCR and Southern blot analyses using human genomic DNA, 27 sets of primers were finally obtained. Thereafter, out of 27 loci, 12 sets of competitive PCR primers were successfully generated (supplementary Table S1). The primer sets for three target loci (*i.e.*, AC4886-158, Z83847-223, and Y978-250) from the 12 sets were generated from unique regions in their flanking sequences. The abundance of each target locus in the nascent DNA fractions relative to genomic DNA was examined by competitive PCR. The data obtained indicated that DNA from the XPD4-C6 locus was as abundant as the *c-myc* origin-derived DNA (Figure 7). Despite somewhat low abundances overall, the DNA abundances of seven loci (*i.e.*, AC4886-158, Z83847-223, XPD1-C5, XPD2-G2, XPD4-C6, XPD4-D3, and XPD5-C6) were more than 4-fold higher than XPD4-F10 (Figure 7). This finding suggests that the early-replicating origins are most likely located close to these loci, and also that the origins can be concentrated effectively by the origin-trapping method using cell synchronization. Enrichment of the origin loci tested from library II showed no statistically significant trends; however, two possible reasons might account for this. First, the poor enrichment observed in library II may have been caused by incidental contamination with broken BrdU-incorporated non-origin fragments during nascent DNA purification. Alternatively, the five loci that exhibited poor abundances may be derived from inefficient origins activated by the cell synchronization treatment. Previous studies have shown that most origins are used in less than 10% of the cell cycles [32,40,41,42,43], suggesting that mammalian origin usage is highly flexible. Altered patterns of replication initiation have also been found in mammalian cells treated with DNA synthesis inhibitors [44] as described later. Therefore, cell synchronization treatment might activate a subset of inefficient origins. In addition, significant differences in the G+C composition and the sequence motif contents were not found between the seven putative origin loci and the other five loci investigated here.

### 2.7. Sequence Analyses of the DNA Clones from Libraries I and II

**Figure 8:**
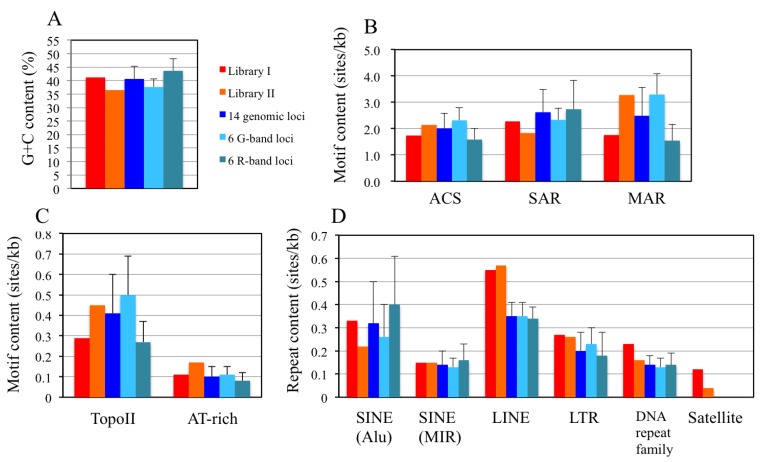
The G+C content and sequence compositions of repeat elements and replication-related motifs in libraries I and II. supplementary Table S5 contains additional information. It should be noted that the human genome sequences used as controls contained no satellite elements. Bars represent the standard deviation of the mean.

The nucleotide sequences of the DNA clones from the two libraries were characterized using bioinformatics to examine the features of their primary structures. We examined the G+C composition of a contiguously assembled sequence from each library, as well as the composition of the repeat sequences, and five replication-related sequences (ACS [45], nuclear scaffold sequences [SAR and MAR] [46,47], topoisomerase II recognition sequences [48], and AT-rich elements). Because several studies [26,27,28,29,49] suggest that replication origins are associated with the nuclear scaffold, the composition of the SAR and MAR sequences was examined. Topoisomerase II sites have been shown to associate with MARs [46]. Furthermore, AT-rich regions have frequently been observed in the replication origins of the fission yeast genome [2,3,4,5] and in about 1% of the human genomic region [14]. In contrast, some studies suggest that human replication origins are associated with G+C-rich regions of the genome [11,19]; hence, the G+C composition and the content of the AT-rich elements were examined. Genome sequences (90–200 kb/locus) from 14 loci including each of the six loci from cytogenetic G- and R-band regions were selected from the human genome and used as controls (supplementary Table S5A). As shown in Figure 8A and supplementary Table S5B, the G+C contents of libraries I and II were 41.2 and 36.5%, respectively. The G+C content of library I is close to the average value of about 41% for the human genome [50]; however, library II has a lower value. The compositions of the repeat sequences and motifs in the libraries had similar values to the mean values of the 14 control loci. However, it should be noted that the compositions of the AT-rich elements (0.17 sites/kb) in library II (Figure 8C), and long interspersed elements (LINEs) in both libraries (0.55 and 0.57 sites/kb in the library I and II, respectively) (Figure 8D), were higher than the corresponding mean values (0.10 for AT-rich elements and 0.35 for LINEs) of the control loci (Figure 8C,D and supplementary Table S5B). In addition, the composition of the MAR sequences (3.27 sites/kb) in library II and the DNA repeat family (0.23 sites/kb) in library I were higher than the corresponding mean values of the control loci (2.48 and 0.14 sites/kb, respectively) (supplementary Table S5B). Although previous studies have suggested that a close association exists between the replication initiation sites and the nuclear scaffold [30], substantial differences in the compositions of the MAR and SAR sequences and topoisomerase II recognition sequences were not found between the sequences in the libraries and the control loci (Figure 8B,C). As for the replication-related motifs, Price *et al.* have reported that a 36-bp consensus sequence supports autonomous replication in human cells [25]; however, we did not detect this sequence in our libraries (data not shown). In addition, a recent genome-wide mapping study of λ exonuclease-resistant short DNAs revealed that human replication origins associate with G-quadruplex motifs [19]. Analysis of our putative origins with the QGRS Mapper program showed that 55.6 and 39.8% of the sequences in library I, and 62.9 and 43.4% of the sequences in library II, contained G4L1-15 and G4L1-7 motifs, respectively (supplementary Table S3). However, it is difficult to make direct comparisons between our data and the data from the genome-wide mapping study because these studies differed in their experimental conditions and the way in which the motif analyses were performed.

The sequence compositions of the two libraries could be clearly distinguished by their G+C content as well as by the composition of some of the motifs and repeat sequences (e.g., MAR, AT-rich elements and Alu repeats). Few Alu sequences and a low G+C content in library II are features generally observed in the G-band region of chromosomes, and are consistent with the corresponding mean values of the six G-band loci (Figure 8A and supplementary Table S5). Nevertheless, library I had a slightly higher G+C content (41%), and its motif compositions were similar to the mean values of the six R-band loci. As was mentioned in the Section 2.6, these differences are likely to reflect the different origin-trapping methods used for preparation of the libraries, especially library II, which is derived from cells treated with hydroxyurea and aphidicolin. Anglana *et al.* reported that depletion of nucleotide pools by hydroxyurea treatment modulated the initiation of replication in Chinese hamster cells [44]. Mesner and colleagues prepared origin-enriched libraries from both log-phase and synchronized human cells and found that the sequence compositions of the resultant libraries differed from each other; hence, they concluded that cell synchronization affects the replication initiation sites [17]. Thus, differences in the features of the sequences from libraries I and II might be explained by the presence of different initiation origins in the synchronized cells that had their nucleotide pools depleted with hydroxyurea.

### 2.8. In Silico Mapping of the Putative Replication Origin Sequences to the Human Genome and Analysis of their Flanking Sequences

**Figure 9:**
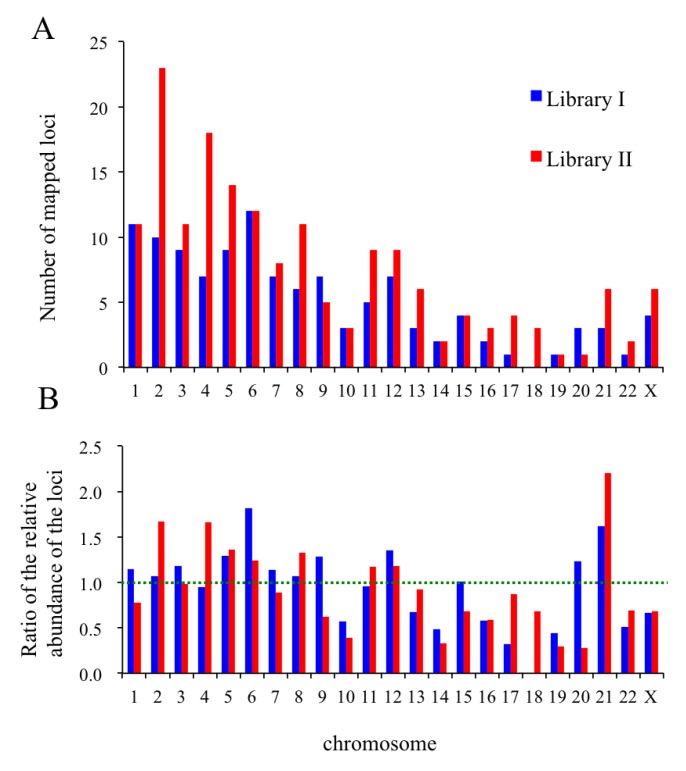
Assignment of the putative replication origin loci from libraries I and II to human chromosomes. (**A**) Histogram showing the number of loci from each library that mapped to individual chromosomes. The number of mapped loci from library I (blue) and II (red) are indicated; (**B**) Ratio of the abundance of mapped loci relative to the chromosome size. A green dotted line depicting a ratio of 1.0 indicates that the abundance of the mapped loci can, in theory, be predicted from the size of the chromosome (supplementary Table S7).

The putative origin sequences from the libraries were further mapped to the human genome using BLAST/BLAT searches. One hundred and seventeen and 172 unique loci from 133 (library I) and 221 (library II) sequences were successfully assigned to their respective genomic locations on human chromosomes (supplementary Table S6A,B). Figure 9A is a histogram of the number of loci that mapped to each chromosome, while the ratios for locus abundance to chromosome size are shown in Figure 9B. The abundances of the loci fluctuate around a ratio of 1.0 (green dotted line, Figure 9B) for more than half of the chromosomes, indicating a correlation between the origin contents and chromosome size. Interestingly, the putative origin loci for chromosomes 6 and 21 from library I and for chromosomes 2, 4 and 21 from library II had ratios over 1.5 (supplementary Table S7). In contrast, loci on chromosomes 17–19 from library I and on chromosomes 10, 14, 19 and 20 for library II were underrepresented in comparison with the values predicted for their associated chromosome sizes (*i.e.*, they had ratios <0.5). In a related genome-wide study using human cells, Besnard *et al.* observed that the distribution of origin peaks had no correlation with chromosome length [19]. We also found no consistent correlations between the G+C compositions and/or the contents of the CpG islands and the abundance of loci mapping to these chromosomes. For example, although chromosome 19 has the highest G+C content (49%) and CpG island composition (19.7 sites per Mb) among the human chromosomes [50], fewer loci from both of the libraries mapped to this chromosome than to any of the other chromosomes (Figure 9A).

**Figure 10:**
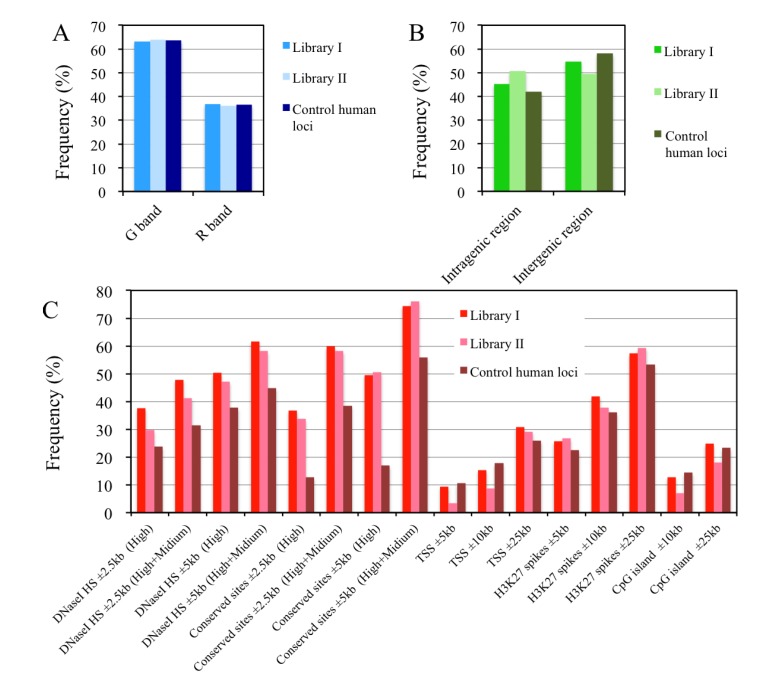
Analyses of the flanking regions of the putative replication origin loci mapped to the human genome. The frequencies with which library I and II loci and 236 loci selected from the human genome (control human loci) mapped to G- or R-band regions (**A**) or to intra- or inter-genic regions (**B**). It should be noted that the ratio of the G- and R-bands in the “Control human loci” does not reflect the actual ratio of the bands in the human genome (see Section 3.9 for further explanation); (**C**) Frequency of loci with functional sites in their flanking regions. Information about human genomic loci was mainly obtained from the UCSC Human Genome Browser [54]. The number of loci with functional sites in their flanking regions was counted within the window sizes indicated. Digital DNaseI hypersensitive sites (DNaseI HS) and mammalian conserved sites were classified as being high (H) or medium (M) based on the strength of the signals. TSS: transcription start site. A 5'-terminus of the predicted genes in the UCSC Genes track was considered a TSS. H3K27Ac peaks: The peak signals of K27-acetylated histone H3. A summary of the data appears in supplementary Table S8, and details of the compositions of the sites for each sequence appear in supplementary Table S6A–C.

Next, we assigned each locus to a chromosome band. As shown in Figure 10A and in supplementary Table S8, more than 60% of the loci from both of the libraries mapped to G-band regions. The results from library II were unexpected because G- and R-band regions are believed to replicate in late and early S phase, respectively [51]. If this hypothesis about chromosome bands and replication timing is correct, the R-band loci should be enriched in the library prepared from the early S phase cells (library II). The discrepancy between previous cytogenetic observations and our results may be partially explainable based on the results from recent studies on chromosome bands. Studies on high-resolution banding and replication timing now suggest that a chromosome band is made up of several domains (or isochores) that differ in their replication timing [52,53]. Thus, a single G-band is likely to contain multiple domains that replicate in early S phase. Consequently, it is possible that some loci from library II are derived from chromosomal domains in the G-band regions.

We examined the flanking regions of the mapped loci in the human genome using the project data from ENCODE [55] in the UCSC Genome Browser [54] to look for associations with the predicted genes (Figure 10B), transcription start sites (TSSs), DNase I hypersensitive sites (DNaseI HSs) suggestive of active chromatin domains, mammalian conserved sequences (indicating important genetic elements such as exons), CpG islands (implicated in regulation of gene expression), and the K27-acetylated histone H3 peaks associated with transcriptionally active chromatins (Figure 10C). To compare the compositions of these sites in the flanking sequences, 236 genomic regions from human chromosomes were selected as control loci. This is in keeping with the same R/G-band ratio of the libraries and where the number of control loci from each chromosome almost corresponds to a chromosome size (supplementary Table S6C). Approximately 50% of the putative origin loci from the libraries tended to be located in the intragenic regions, which compared with 42% in the corresponding control loci (Figure 10B). Increased numbers of conserved sites (mainly exons) were detected in the flanking regions of the loci from both libraries in 5 and 10 kb-windows compared with those from the control loci (Figure 10C). In a recent study on origin mapping (using human HeLa and normal lymphoblastoid cell lines), Mesner *et al.* [17] showed that significant fractions (ca. 50%) of the origins resided within the bodies of genes, as was also observed in this study. Valenzuela *et al.* [18] and Cadoret *et al.* [11] also observed preferential localization of replication origins at evolutionarily conserved sequences in about 1% of the human genome in their origin mapping studies. In the recent genome-wide origin mapping study by Besnard *et al.* [19], 57.3% of the origins were associated with genes (promoters, exons and introns), but the majority (45.9%) were found in introns. In the present study, DNase I HSs were also more frequently detected in the flanking sequences (in 5 and 10 kb-windows) of the putative origin loci than in the control loci (Figure 10C). These data strongly suggest a tendency for origin loci to be located in gene (exon)-rich intragenic regions containing open chromatin domains.

By contrast, TSSs, CpG islands, and K27-acetylated histone H3 peaks in the flanking regions of the 10, 20, and 50 kb windows, were similarly observed in both the putative origin loci and the control loci (Figure 10C), suggesting that the majority of the mapped loci are not located near the 5'-upstream regions of the genes. Interestingly, a recent study on genome-wide origin mapping indicated that less than 18% of the origins were associated with TSSs and CpG islands in the human genome [19], a value that is in keeping with our own observations. Although replication origins have been shown to be closely associated with CpG islands and/or TSSs (including promoters in the 5'-upstream regions of the genes) in previous studies with specific mammalian origins [56,57] and the comprehensively mapped origins in fly [15], mouse [12,15] and humans [11,14,18], we do not have any evidence that can account for the discrepancy between these findings and those of our own study. The potential cryptic origins identified in library II of the present study may have arisen from treatment with hydroxyurea. The majority of the origins in the libraries might represent subsets of the origins that are located in the intragenic regions of the genome, rather than in the 5'-upstream regions of the genes.

## 3. Experimental Section

### 3.1. Cell Culture

The human nucleotide excision repair-deficient cell line GM8207 (XP6BE[SV40]) from the XP-D female patient [35] was used in this study. Cells were maintained in Dulbecco’s modified Eagle’s medium (DMEM) supplemented with 10% dialyzed fetal calf serum (dFCS, Sigma, St. Louis, MO, USA or Gibco BRL, Gaithersburg, MD, USA) and 5 μg/mL gentamicin (Sigma) at 37 °C in a CO_2_ incubator. The cells used in the experiments were free of *Mycoplasma* contamination.

### 3.2. UV Irradiation Experiments

Exponentially growing GM8207 cells were cultured in 15-cm-diameter culture dishes with 20 mL of DMEM containing 10% dFCS (*i.e.*, growth medium). The cells were synchronized by incubating at 37 °C for 10 h in the presence of 1 μg/mL aphidicolin (Wako, Osaka, Japan). After washing the cells with 20 mL of warm Ca^2+^ and Mg^2+^-free phosphate-buffered saline (PBS(−)), the cells were cultured in the growth medium for a further 10 h in the presence of 0.03 μg/mL colcemide (Gibco BRL). Mitotic cells transferred into conical tubes by gentle pipetting were collected by centrifugation at 1,300 rpm for 5 min at room temperature. After washing the cells with 20 mL of warm DMEM (twice), they were suspended in growth medium. Approximately 1.5 × 10^6^ cells were inoculated into a 6-cm culture dish with 5 mL of warm growth medium and cultured for a further 5 h at 37 °C. Cells synchronized in S phase were gently washed by soaking in 5 mL of warm PBS(−) with aspiration. Warm PBS(−) (0.4 mL) was then added to the dish and the cells were simultaneously irradiated using short wave ultraviolet light (254 nm, 500 J/m^2^) under a UV lamp (UVGL-58, UVP Inc., Upland, CA, USA). After aspirating with PBS(−), 2 mL of warm growth medium containing 10 μM fluorodeoxyuridine (FUdR: Sigma) was added. After a 30-min culture at 37 °C, the cells were incubated further with 75 μM 5-BrdU (Sigma) for 15 min to label the newly synthesized DNA. In parallel, the cells in one plate were labeled with 10 μCi/mL of [^3^H]TdR, (118 Ci/mmoL, Amersham, Piscataway, NJ, USA) to monitor the size of the labeled DNAs, as reported previously [58]. The labeling reaction was terminated by addition of sodium azide at a final concentration of 2 mM with chilling on ice, after which the cells were washed with ice-cold PBS(−) and fixed by addition of 0.7 mL of cold ethanol. Cells fixed in this way were scraped from the dish, transferred into Eppendorf tubes and collected by centrifugation. The cells were suspended in 0.7 mL of cold ethanol and stored at −20 °C in the dark for purification of the BrdU-incorporated nascent DNAs. For monitoring the influence of UV irradiation on DNA synthesis, the cells were irradiated with the UV doses indicated above, and pulse labeling of the DNA with [^3^H]TdR was performed in FUdR-containing growth medium for 15 min then terminated as described above. For the pulse-chase experiments, after a 15-min pulse, the radioactive growth medium was replaced with 4 mL of warm growth medium containing 10 mM TdR, after which the cells were incubated at 37 °C for the time periods indicated above, and then harvested for alkaline sucrose density gradient centrifugation.

### 3.3. Chemical Crosslinking Experiments

DNA crosslinking with Trioxsalen and long-wave UV light was performed according to a previously reported procedure by Russev and Vassilev [33] with the modifications described below. DNA synthesizing GM8207 cells were used for analyzing the effects of crosslinking in DNA replication. Cells (1.5 × 10^6^) synchronized in S phase were prepared by aphidicolin treatment for 15 h followed by a 2-h release. After removing the medium from a 6-cm dish by aspiration and washing the cells with PBS(−), 3 mL of PBS(−) with 15 μL of Trioxsalen (Sigma, 0.5 mg/mL in 50% ethanol) was added and the dish placed on ice for 2 min. The cells were irradiated under a high pressure mercury lamp (BHRF500WH, Iwasaki Electronics, Tokyo, Japan) at a distance of 10 cm (lid-to-lamp) using the conditions indicated above. The reagents were removed by aspiration and washing with 5 mL of PBS(−), after which the cells were incubated in 2 mL of the growth medium at 37 °C for 30 min. The experimental conditions for the pulse-label and chase are described in Section 3.2. For nascent DNA preparation after DNA crosslinking, logarithmic growing GM8207 cells were used. Crosslinking was performed by 2-min irradiation (twice), followed by incubation in 2 mL of growth medium at 37 °C for 15 min, after which the cells were labeled with BrdU for 20 min. Labeling reactions were terminated and the cells were fixed as described in Section 3.2.

### 3.4. BrdU-Labeling of Nascent DNA Synthesized in Early S Phase

GM8207 cells were arrested in early S phase with replication inhibitors and labeled with BrdU to prepare nascent DNAs from the early-firing origins. Briefly, exponentially proliferating cells (≈50% confluent) in a 15-cm dish with 20 mL of growth medium were incubated with 1.25 μg/mL of aphidicolin at 37 °C for 12 h. The cells were released by removing the medium and successive washing with 20 mL of warm DMEM (twice), and cultured in 15 mL of warm growth medium containing 0.03 μg/mL colcemide for 12 h in the dark. Mitotic cells were collected into conical tubes and washed in 40 mL of warm DMEM twice as described in Section 3.2. Cells (2 × 10^6^) were inoculated and cultured in a collagen-coated 6-cm dish with 3 mL of warm growth medium containing 5 μg/mL aphidicolin and 1.5 mM hydroxyurea at 37 °C for 5 h. Thereafter, the cells were continuously labeled with 40 μM BrdU at 37 °C for 4 h in the dark and harvested as described in Section 3.2.

### 3.5. Purification of BrdU-Labeled DNA

BrdU-labeled DNAs in the fixed cells were purified by fractionation using alkaline sucrose density gradient centrifugation and successive immunoprecipitation with an anti-BrdU antibody. Cells were handled under dim light or in the dark. Cells fixed in ethanol were pelleted, suspended and washed in 1 mL of ice cold PBS(−) by centrifugation at 5,000 rpm for 2 min (4 °C). The cells were gently resuspended in 0.3 mL of TNE (10 mM Tris-HCl, pH 7.5, 0.1 M NaCl, 1 mM EDTA, pH 8.0) and incubated at 37 °C overnight in the presence of 1% SDS and 0.3 mg/mL proteinase K (Merck, Darmstadt, Germany). After gentle extraction by rotation with phenol-chloroform for 20 min and successive centrifugation at 12,000 rpm for 5 min, the DNA in the aqueous layer was precipitated with 0.25 M KCl and 2.5 volume of ethanol. After washing with 1 mL of 80% ethanol by centrifugation at 12,000 rpm for 10 min, the pellet was air-dried and dissolved in 50 μL of autoclaved distilled water at 4 °C for 5 h. The DNA solution was loaded onto 0.2 mL of alkaline lysis solution (0.5 M NaOH, 0.7 M NaCl, 10 mM EDTA, and 2% sodium *N-*lauroylsarcosinate) overlaid on top of 35 mL of a linear sucrose gradient (5 to 25%) with 0.2 mL of a 60% sucrose cushion in the solution containing 0.3 M NaOH, 0.7 M NaCl, 10 mM EDTA, 0.1% sodium *N-*lauroylsarcosinate. The samples were allowed to stand at 4 °C for 7 h in the dark then centrifuged at 25,000 rpm for 16 h in a Beckman SW28 rotor. Fractions (1 mL each) were carefully collected from the top of the tube. Fractions containing newly synthesized DNA were pooled and each 8 mL of a fraction transferred into a silane-coated centrifugation tube and mixed with ammonium acetate, ethanol. Twenty micrograms of glycogen (Boehringer, Ingelheim, Germany) was used as a carrier. The tube was left at −80 °C for 1 h and the DNA precipitated by centrifugation at 23,000 rpm for 30 min in a SW28 rotor. The DNA was washed with 1 mL of 80% ethanol, air-dried and dissolved in 40 μL of TE buffer (10 mM Tris-HCl, 1 mM EDTA, pH 8.0). For DNA preparation from UV irradiated cells, the fixed cells were washed with PBS(−) and suspended in 50 μL of PBS(−). Samples were directly loaded onto the alkaline lysis solution and left at 4 °C for 8 h before the following centrifugation step. The distribution of nascent DNA in the fractions was monitored by a parallel centrifugation of [^3^H]TdR-labeled cells as described in the above sections. [^32^P]-labeled linear pBluescript DNA (*Xho*I cut) was used as a 3 kb size marker in a parallel tube. The distribution of the DNA molecules in a gradient was also confirmed by a parallel centrifugation with a [^32^P]-labeled 1 kb DNA ladder (Gibco BRL) and successive alkaline gel electrophoresis. Approximately 1–2 mg of purified DNA was used for further immunopurifications. Purified DNA was gently mixed at 4 °C for 1 h in 0.3 mL of binding buffer (20 mM Tris-HCl, pH 8.0, 50 mM NaCl, 1 mM EDTA, 0.1% NP-40) containing 0.2 mL of anti-BrdU monoclonal antibody (clone B44, Becton and Dickinson, San Jose, CA, USA). Then, 20 μL of protein A/G-coated latex beads (Interfacial Dynamics Corp., Portland, OR, USA) were added and the solution incubated at 4 °C for a further 1.5 h in a rotator. Prior to the experiments, the binding capacity of the BrdU-labeled single-stranded DNA and the negligible nonspecific DNA binding of the latex beads were examined. The beads were collected by centrifugation, successively washed with 0.3 mL of binding buffer (once) followed by autoclaved distilled water (at least three times), then suspended in 0.1 mL of TE buffer containing 0.2% SDS and 0.5 mg/mL proteinase K. After incubation at 37 °C for 1.5 h and phenol-chloroform extraction, the DNA was purified by ethanol precipitation with 2 μg of glycogen.

### 3.6. Cloning of BrdU-Labeled DNA

Purified BrdU-labeled single-stranded DNA was modified through addition of a poly dC tail at the 3'-end with TdT (Gibco BRL) and dCTP according to the manufacturer’s instructions. Reactions were terminated by phenol-chloroform extraction and the DNA collected by ethanol precipitation. The complementary strand of the poly dC-tailed single-stranded DNA was synthesized with DNA polymerase and an oligo dG primer. Synthesis of single-stranded DNA from UV irradiated cells was performed with Sequenase™ (version 2, USB, Cleveland, OH, USA). After incubation with DNA and 50 pmoL of the 5'-phosphorylated oligo dG/I primer (5'-GGIIGGGIIGGGIIGGGIIGG-3') at 37 °C for 30 min in a 50 μL reaction mixture, 40 units of Sequenase and the recommended concentrations of dithiothreitol and dNTPs were added and the reaction incubated for 60 min. For nascent DNA from early S phase cells, rTaq DNA polymerase (Toyobo, Tokyo, Japan) was used for DNA synthesis. The 50 μL reaction containing the DNA sample, 10 units of rTaq polymerase, 125 pmoL of the 5'-phosphorylated oligo dG_12–18_ primer (Pharmacia, Uppsala, Sweden), and other components were incubated at 65 °C for 4 min, according to the manufacturer’s instructions. DNA synthesis in each reaction was monitored in parallel reactions containing [^32^P]dCTP and successive 1.3% alkaline agarose gel electrophoresis and autoradiography. After phenol-chloroform extraction and ethanol precipitation, DNA ends were blunted using a DNA blunting kit (Takara Bio, Otsu, Shiga) and the 5' ends of the DNA were phosphorylated with T4 polynucleotide kinase (Toyobo or Gibco BRL), according to the manufacturer’s instructions. DNA purified by phenol-chloroform extraction and ethanol precipitation was ligated to a DNA adaptor (UniAmp, Clontech, Mountain View, CA, USA) with T4 DNA ligase (Ligation high, Toyobo). Next, the adaptor-ligated DNAs were PCR amplified with an adaptor-specific primer. *Eco*RI and *Xho*I adaptors, and ΔTth DNA polymerase (Toyobo) and *Ex Taq* DNA polymerase (Takara Bio) were used for adaptor-ligation and PCR amplification of DNA from the UV irradiated and synchronized cells, respectively. After 40 cycles of amplification, the products were separated on a 2% agarose gel. Approximately 0.5–3 kb DNA molecules were purified from the gel by electro-elution followed by ethanol precipitation. The DNA ends were blunted and phosphorylated as described above. The resultant DNA was ligated to *Sma*I-digested and dephosphorylated pUC19 DNA and used to transform *E. coli* DH5α cells. Approximately 500–700 clones derived from the UV-irradiated and the synchronized cells were isolated and designated libraries I and II, respectively, and thereafter used for sequence analyses.

### 3.7. DNA Sequencing

Preparation and sequencing of plasmid DNA, followed by editing and assembly of the sequences were performed as described previously [45]. Briefly, nucleotide sequences from insert DNAs were read in both directions by an automated DNA sequencer (type 373A, ABI, Foster City, CA, USA). The large DNA sequences in some of the plasmids were sequenced using a SequiTherm™ long-read cycle sequencing kit (Epicentre Technologies, Madison, WI, USA) and analyzed using a long-read sequencer (type 4000L, LICOR, Lincoln, NE, USA). Sequence IDs that are preceded by “2D_” and “D1_–D5_” correspond to clones prepared from libraries I and II, respectively (supplementary Table S3). Non-human DNAs, mitochondrial DNAs, and human DNAs less than 200 bp were removed from the libraries. Nucleotide sequences were deposited in the DNA Data Bank of Japan (accession numbers: AB761625 to AB761978).

### 3.8. Competitive PCR

Competitive PCR was performed as a quantitative assay of the target sequences in the purified DNA fractions. Genomic DNA used as a control was prepared from confluent GM8207 cells in stationary phase. The procedure was conducted according to Diviacco *et al.* [36]. Briefly, a pair of outer PCR primers and two inner primers (with 20-bp tails at the 5'-ends), were prepared for each locus for amplification of the target DNA and the 20-bp larger competitor DNA. The details of the primers used in the study are shown in supplementary Table S1. Competitor DNA was amplified as described in reference [27] and purified from the gel for DNA quantification. Some competitor DNAs were connected by PCR and their products were cloned into pT7Blue DNA (Novagen, Darmstadt, Germany) to use as competitor DNA. The 10-μL reaction mixture contained 10 mM Tris-HCl, pH 8.3, 50 mM KCl, 1.5 mM MgCl_2_, 0.2 mM each dNTP, 20 pmoL of each primer, 0.5 unit of rTaq DNA polymerase, and 2–5 ng of template DNA with either 40, 130, 400, 1,200, 3,600, 10,800, or 32,400 molecules of the competitor DNA. In some reactions, 0.1 unit of Perfect Match (Stratagene, La Jolla, CA, USA) was added. Competitive PCR was performed for 40–50 cycles at 94 °C for 20 s, 55–65 °C for 30 s, and 72 °C for 60 s in a GeneAmp PCR System 9600 thermal cycler (Perkin-Elmer, Foster City, CA, USA). Heat denaturation of the DNA at 94 °C as an initial PCR step was omitted. PCR products were separated by electrophoresis on a 10% acrylamide/15% glycerol gel, and then stained with ethidium bromide. Gel band images were acquired with a CCD camera and analyzed by NIH Image for quantitative analysis.

### 3.9. Sequence Data Analyses and In Silico Mapping to the Human Genome

G+C content determination and prediction of replication-related sequence motifs (*i.e.*, autonomously replicating sequence core sequence [ACS] [45], nuclear scaffold associated region [SAR] [47], nuclear matrix associated region [MAR] [46], topoisomerase II recognition consensus sequence [48], and a 36-bp human ARS [25]) were all performed using Genetyx MAC software (version 16, Genetyx Corp., Tokyo, Japan) as described previously [45]. The number of sequence motifs in a sequence was counted after removing redundant sites. Detection of repeat sequences, low complexity sequences and simple repeat elements including AT-rich elements was performed by the RepeatMasker program [59] under the default setting (AB-Blast engine) via the internet [60]. A single sequence that was assembled from all of the library sequences was used for G+C content and sequence motif content analyses. The sites of G-quadruplex sites with loop lengths of 1–7 nucleotides (G4L1-7) or with loop lengths of 1–15 nucleotides (G4L1-15) in their sequences were obtained using the QGRS Mapper program [61] (with the following setting: Max length, 30; Min-G-group, 2; indicated loop size) via the internet [62]. *In silico* mapping of the putative origin sequences to the human genome and assignment of the chromosome bands were performed by BLAST searches [63] against the *Homo sapiens* build 37.3 genome database (updated on August 2011) in the National Center for Biotechnology Information (NCBI) [64] and by BLAT searches [65] against the human genome assembly data (GRCh37/hg19) in the UCSC Genome Browser [66] and the Ensemble browser [67,68]. Information about the flanking genome sequences, including the predicted genes (the UCSC Genes track), DNase I hypersensitive sites, K27-acetylated histone H3 peaks, CpG islands, and conserved regions among vertebrates was obtained from the UCSC Genome Browser. For analysis of the nucleotide sequence features, 14 sequences from the human genome (each of the six sequences from R- and G-band regions and two from the boundary regions of the bands) were selected as controls; these had comparable G+C content (ca. 36%) to the sequences tested from the libraries (supplementary Table S5). Two hundred and thirty-six loci from the human genome were also selected for analysis of their flanking sequences; this was in keeping with the ratio of the length of each of the chromosomes, as well as with the comparable R/G-band ratio of the loci tested from the libraries (supplementary Table S7C).

## 4. Conclusions

The aim of this study was to isolate and clone nascent DNAs from replication forks that had arrested near origins of replication in the human genome. We characterized the effects of UV radiation and chemical crosslinking during DNA synthesis in human DNA repair-deficient cells and used UV radiation to clone the nascent DNAs from the origins in the exponentially proliferating cells (Figure 1A). In parallel, we cloned nascent DNAs from cells synchronized in early S phase in the presence of BrdU, aphidicolin and hydroxyurea. Under these conditions, replication forks that initiate in early S phase will arrest and BrdU-labeled nascent DNAs can accumulate around the origins (Figure 1B). Enrichment of the origin-derived DNAs by the UV-mediated origin-trapping method (Figure 6) and the potential origin activities of seven loci identified by the cell synchronization method (Figure 7) were confirmed by use of a quantitative competitive PCR assay, suggesting that successful concentration of the origin-derived DNAs was achieved by these origin-trapping methods. We also showed enrichment of the origin-derived fragments using Trioxsalen-mediated chemical crosslinking (Figure 5, Figure 6), which has previously been reported although the enriched DNAs from authentic origins were not identified in the mammalian cells in that study [34]. Thus, in addition to UV-radiation, chemical crosslinking may be a useful tool for trapping origins of replication. Sequence analyses of the isolated putative origins revealed distinct sequence compositions for each of the two libraries; this strongly suggests that different origins were isolated by each of the two origin-trapping methods. In the analysis of the flanking sequences, we showed that the putative origin loci from the libraries tended to be located within genes, including evolutionally conserved sites, as reported by others [11,17,18,19]; however, we failed to find any significant association of the loci with specific functional sites (e.g., CpG islands and TSSs) for transcriptional regulators. Although we do not have any evidence that could account for the discrepancy between our observations and those of others on specific functional sites, it is interesting that a recent genome-wide origin mapping study using human cells revealed that a limited fraction of the origins were associated with CpG islands and TSSs [19]. Several lines of evidence indicate that there is flexible usage of replication origins as well as the existence of efficient and inefficient origins (reviewed in [20]). In fact, in two independent studies on origin mapping using the same method (purification of λ exonuclease-resistant DNAs and microarray platforms) and the same cell lines (independent sub-clones of HeLa cells) [11,14], less than 14% of the origins identified were overlapping, suggesting that only a subset of the origins can be detected, even when the same methods are used. Taken together, these findings suggest the possibility that a subset of replication origins (including the putative origins in our study) associate poorly with TSSs and CpG islands.

We found that there are some potential issues to be resolved in our origin-trapping procedures. As shown in Figure 7, five out of 12 loci isolated from the early S phase cells exhibited poor origin activities in the competitive PCR experiment. In addition, although it has been suggested (in previous studies) that regions within R-bands preferentially replicate during early S phase, a relatively large fraction of G-band-derived sequences were identified in library II from the early S phase cells (Figure 10A). These sequences suggest the possibility of potential contamination from DNAs derived from non-origin regions in the libraries and/or from the firing of cryptic (or inefficient) origins by stringent treatments with replication inhibitors to induce cell cycle arrest, as observed in other studies [17,44]. Although genome-wide analyses of replication origins have been performed in mammalian cells whose genomes have already been sequenced, the origin-trapping methods with UV radiation or crosslinking described herein will be useful for obtaining structural information at the sequence level about the regions near the origins in eukaryotes for which no genomic sequences exist.

## Supplementary Files

Supplementary File 1 Supplementary (ZIP, 213 KB)
