# Supplementary material for: Trapping DNA Replication Origins from the Human Genome
Source: Genes (Basel). 2013 Apr 17;4(2):198–225. doi: 10.3390/genes4020198 (PMC3899975; doi:10.3390/genes4020198)
Supplement: Supplementary File 1 — Supplementary (ZIP, 213 KB) [file genes-04-00198-s001.zip › Table S2.doc]

**Table S2.** Competitive PCR assay for assessing the relative abundance of DNA from *c-myc*, *lamin* B2, β-globin_40k, and sWXD1449 loci.

| Target locus | Number of competitor molecules for 50% competition | | | N/G × 100 | Relative abundance (fold) to β-globin_40k |
| --- | --- | --- | --- | --- | --- |
|  | Nascent DNA (N) | Genomic DNA (G) | |  |  |
| Exp. 1 (nascent DNA from UV irradiated cells) | | | | | |
| *lamin* B2 origin | 128 | | 480 | 26.7 | 9.2 |
| β-globin_40k | 78 | | 2700 | 2.9 | 1.0 |
| sWXD1449 | 250 | | 4400 | 5.7 | 2.0 |
| Exp. 2 (nascent DNA from UV irradiated cells) | | | | | |
| *c-myc* origin | 914 | | 3000 | 35.2 | 8.2*^a^* |
| sWXD1449 | 180 | | 4200 | 4.3 | 1.0 *^a^* |
| Exp. 1 (nascent DNA from crosslinked cells) | | | | | |
| *c-myc* origin | 1960 | | 2600 | 75.4 | 17.0 |
| *lamin* B2 origin | 440 | | 480 | 91.7 | 20.6 |
| β-globin_40k | 120 | | 2700 | 4.4 | 1.0 |
| sWXD1449 | 115 | | 4400 | 2.6 | 0.6 |
| Exp. 2 (nascent DNA from crosslinked cells) | | | | | |
| *c-myc* origin | 1730 | | 970 | 178.4 | 11.5 |
| *lamin* B2 origin | 630 | | 290 | 217.2 | 14.1 |
| β-globin_40k | 400 | | 2590 | 15.4 | 1.0 |
| sWXD1449 | 100 | | 510 | 19.6 | 1.3 |

*^a^* Relative abundance (fold) to sWXD1449.
