# Supplementary material for: Trapping DNA Replication Origins from the Human Genome
Source: Genes (Basel). 2013 Apr 17;4(2):198–225. doi: 10.3390/genes4020198 (PMC3899975; doi:10.3390/genes4020198)
Supplement: Supplementary File 1 — Supplementary (ZIP, 213 KB) [file genes-04-00198-s001.zip › Table S4.doc]

**Table S4.** Relative abundance of nascent DNAs from 12 loci from library II by competitive PCR assay.

| **Target locus**  **(Derived sequence ID)** | **Number of competitor molecules for 50% competition** | | **N/G × 100** | **Relative abundance (fold) to XPD4-F10** |
| --- | --- | --- | --- | --- |
|  | **Nascent DNA (N)** | **Genomic DNA (G)** |  |  |
| *c-myc* origin | 140 | 150 | 93 | 7.2 |
| AC4886-158 (D1_B01) | 81 | 140 | 58 | 4.5 |
| Z83847−223 (D1_H04) | 70 | 101 | 69 | 5.3 |
| Y978-250 (D2_C04) | 37 | 100 | 37 | 2.8 |
| XPD1-C5 (D1_C05) | 26 | 110 | 24 | 1.8 |
| XPD1-H3 (D1_H03) | 55 | 80 | 69 | 5.3 |
| XPD2-G2 (D2_G02) | 31 | 60 | 52 | 4.0 |
| XPD3-C2 (D3_C02) | 25 | 105 | 24 | 1.8 |
| XPD4-C6 (D4_C06) | 104 | 105 | 99 | 7.6 |
| XPD4-D3 (D4_D03) | 110 | 190 | 58 | 4.5 |
| XPD4-F10 (D4_F10) | 90 | 700 | 13 | 1.0 |
| XPD5-C6 (D5_C06) | 74 | 104 | 71 | 5.5 |
| XPD5-F3 (D5_F03) | 38 | 190 | 20 | 1.5 |
