# Supplementary material for: Trapping DNA Replication Origins from the Human Genome
Source: Genes (Basel). 2013 Apr 17;4(2):198–225. doi: 10.3390/genes4020198 (PMC3899975; doi:10.3390/genes4020198)
Supplement: Supplementary File 1 — Supplementary (ZIP, 213 KB) [file genes-04-00198-s001.zip › Table S8.doc]

**Table S8.** Analyses of the flanking regions of the putative replication origin loci mapped to the human genome.

|  | **Library I** | | **Library II** | | **Control human loci** | |
| --- | --- | --- | --- | --- | --- | --- |
|  | **No.** | **%** | **No.** | **%** | **No.** | **%** |
| No. of mapped loci | 117 | 100.0 | 172 | 100.0 | 236 | 100.0 |
| G band | 74 | 63.2 | 110 | 64.0 | 150 | 63.6 |
| R band | 43 | 36.8 | 62 | 36.0 | 86 | 36.4 |
| Intragenic region | 53 | 45.3 | 87 | 50.6 | 99 | 41.9 |
| Intergenic region | 64 | 54.7 | 85 | 49.4 | 137 | 58.1 |
| Functional sites in the flanking region | | | | | | |
| DNaseI HS ±2.5 kb (H) | 44 | 37.6 | 51 | 29.7 | 56 | 23.7 |
| DNaseI HS ±2.5 kb (H+M) | 56 | 47.9 | 71 | 41.3 | 74 | 31.4 |
| DNaseI HS ±5 kb (H) | 59 | 50.4 | 81 | 47.1 | 89 | 37.7 |
| DNaseI HS ±5 kb (H+M) | 72 | 61.5 | 100 | 58.1 | 106 | 44.9 |
| Conserved sites ±2.5 kb (H) | 43 | 36.8 | 58 | 33.7 | 30 | 12.7 |
| Conserved sites ±2.5 kb (H+M) | 70 | 59.8 | 100 | 58.1 | 91 | 38.6 |
| Conserved sites ±5 kb (H) | 58 | 49.6 | 87 | 50.6 | 89 | 16.9 |
| Conserved sites ±5 kb (H+M) | 87 | 74.4 | 131 | 76.2 | 132 | 55.9 |
| TSS ±5 kb | 11 | 9.4 | 6 | 3.5 | 25 | 10.6 |
| TSS ±10 kb | 18 | 15.4 | 15 | 8.7 | 42 | 17.8 |
| TSS ±25 kb | 36 | 30.8 | 50 | 29.1 | 61 | 25.8 |
| H3K27Ac peaks ±5 kb | 30 | 25.8 | 46 | 26.7 | 53 | 22.5 |
| H3K27Ac peaks ±10 kb | 49 | 41.9 | 65 | 37.8 | 85 | 36.0 |
| H3K27Ac peaks ±25 kb | 67 | 57.3 | 102 | 59.3 | 126 | 53.4 |
| CpG island ±10 kb | 15 | 12.8 | 12 | 7.0 | 34 | 14.4 |
| CpG island ±25 kb | 29 | 24.8 | 31 | 18.0 | 55 | 23.3 |

Note: Information about the human genomic loci was mainly obtained from the UCSC Human Genome Browser (GRCh37/hg19). Numbers of loci with functional sites in the flanking regions were counted within the indicated window size. Digital DNaseI hypersensitive sites (DNaseI HS) and mammalian conserved sites were classified as being high (H) or medium (M) based on the strength of the signals. TSS: transcription start site. A 5'-terminus of the predicted genes in the UCSC Genes track was considered a TSS. H3K27Ac peaks: The peak signals of K27-acetylated histone H3. Details of the compositions of the sites for each sequence appear in supplementary Tables S6A-S6C.
